# Understanding the visual search strategies of expert and novice coaches in futsal set pieces

**DOI:** 10.3389/fpsyg.2024.1390536

**Published:** 2024-07-08

**Authors:** Manuel Rodrigues, Nuno Leite, João N. Ribeiro, Jaime Sampaio, Duarte Araújo, Bruno Travassos

**Affiliations:** ^1^Department of Sports Sciences, Exercise and Health, Universidade Trás-os-Montes e Alto Douro, Vila Real, Portugal; ^2^Research Center in Sports Sciences, Health Sciences and Human Development, Centro de Investigação em Desporto, Saúde e Desenvolvimento Humano (CIDESD), CreativeLab Research Community, Vila Real, Portugal; ^3^Portugal Football School, Federação Portuguesa de Futebol, Oeiras, Portugal; ^4^Department of Sport Sciences, Universidade da Beira Interior, Covilhã, Portugal; ^5^Polytechnic Institute of Guarda, School of Education, Communication and Sports, Guarda, Portugal; ^6^SPRINT Sport Physical Activity and Health Research and Innovation Center, Viana do Castelo, Portugal; ^7^Centro Interdisciplinar de Performance Humana – Organization (CIPER), Faculty of Human Motricity, Universidade de Lisboa, Cruz Quebrada, Portugal

**Keywords:** expertise, visual perception, coaching, team sports, set pieces, decision-making

## Abstract

**Introduction:**

This study aimed to describe the fixation location and the time of the longer fixation of expert and novice futsal coaches before the ball was in play in futsal set pieces.

**Methods:**

A total of 10 experts (ages 48 ± 5) and 10 novice coaches (ages 40 ± 7) participated in the study. They observed that 38 video clips were created to mimic the attack and defensive set-piece moments of the game. Data were collected in a standardized video analysis task using the pupil invisible eye tracker and processed through the pupil cloud platform. The Mann–Whitney test was conducted to evaluate differences in gaze duration between game moments (attack and defense set pieces) and groups (expert vs. novice). Gaze duration was also compared for gaze location between groups. For further comparisons, the game moments (attack and defense set pieces) and the gaze location were summarized in two-dimensional graphics using correspondence analysis.

**Results and discussion:**

The results revealed higher values of gaze duration for attack and defense set pieces for the group of experts than for novices. When considering gaze duration, expert coaches had higher values than novices for the attacker 3, defender 3, barrier 1st, and barrier 2nd gaze locations. The correspondence analysis showed different strategies of visual search and, consequently, gaze locations for attack and defense set pieces. In particular, there was different correspondence for free kicks between the level of expertise and gaze location, while corner and sideline kicks revealed some correspondence between the groups and the gaze location. In free kicks, coaches should be particularly concerned about the relationship between attacker and defender three and the barrier 1st and 2nd line positions. In corner and sideline kicks, coaches should be particularly aware of the relationship between attackers' and defenders' positions.

## Introduction

Futsal, as a team sport, is characterized by its high level of unpredictability, based on constant alternations in the pace of play and possession of the ball. In this context, different game moments are characterized by specific game dynamics, forcing players and coaches to pick up different visual information to prospectively understand the best individual and collective possibilities for actions (Travassos et al., 2012; Rodrigues et al., 2024). For example, previous research reported differences in the visual search strategies and, consequently, in the visual information used by coaches to characterize the attack, defense, and set pieces in game moments (Rodrigues et al., 2024). In particular, the set pieces or “dead ball situations” (Hüttermann et al., 2018), game moments in which the ball is stationary, revealed specificities in the visual information used by futsal coaches in comparison with the regular attack or defense game moment (Rodrigues et al., 2024). Interestingly, the set pieces are a dangerous game moment in which the ball starts stationary, and there are movements of attackers and defenders before the ball is in play to open passing and shooting lines. Previous research showed that the set pieces in the game moment in futsal account for ~25% of all shots made by a team during the game, and in the 2010 European futsal championship, the Portuguese team scored 46% of their goals with set pieces (Leite, 2012). The percentage of successful set pieces seems to be one of the variables that best distinguish winning teams from losing/drawing teams (Santos et al., 2020), requiring further attention from coaches for its preparation.

Within this scenario, since the visual information of the game is circumstantial to each game moment, the coach's knowledge and attunement to the relevant information (i.e., the capacity to perceive the relevant information according to each game moment) is paramount to shaping and guiding their behavior (Wood et al., 2023). In line with the ecological dynamics approach, the coaches' ability to understand game dynamics and support decision-making depends on their perceptual abilities to explore each game moment, extracting the most informational variables (Araújo et al., 2009). Thus, analyzing the coaches' visual search strategies in specific game moments can play an important role in further understanding how coaches explore the game to understand players' and teams' possibilities for action (Roca et al., 2011; Silva et al., 2013).

Mann et al. (2007) suggested that in sports, experts tend to capture relevant information from the environmental context with greater precision than novice coaches. The analysis of gaze behavior using variables, such as the number of fixations, their duration, and location (Lebeau et al., 2016), has sought to distinguish the visual search strategies of different levels of expertise among players and coaches. However, variables including the location of fixations and the quiet eye duration, i.e., the final fixation or tracking gaze at a specific location for a minimum of 100 ms, before movement initiation (Vickers, 2016), have emerged as variables that consistently distinguish experts from novices (Mann et al., 2007; Klostermann and Moeinirad, 2020). Previous research showed that a longer gaze fixation at specific locations and for an optimal amount of time prior to movement proves to be a characteristic of expert players (Vickers, 2016; Klostermann and Moeinirad, 2020). Although considering that coaches do not execute a motor skill in the same way as athletes, in futsal set pieces, it is paramount to understand the specific locations of the longer gaze fixation before the ball is in play to understand the information that supports the anticipation of set piece possibilities for an action (Hüttermann et al., 2018). In fact, the analysis of the information used before the ball is in play could help to understand the anticipation strategies followed by coaches and the sources of information that guide their decision-making (Mann et al., 2007).

To the best of our knowledge, no study with coaches has analyzed the visual search strategies, particularly the longer fixation at a particular location, before the ball is in play in set pieces. Thus, this study aimed to describe the fixation location and the time of the longer fixation of expert and novice futsal coaches before the ball is in play in futsal set pieces. It was hypothesized that expert coaches present longer fixations at different locations than novice coaches.

## Methodology

### Sample

A total of 10 expert (mean age 48 ± 5 years and years of experience 18 ± 4) and 10 novice (mean age 40 ± 7 years and years of experience 1 ± 1) futsal coaches participated in the study. The expert coaches had at least 10 years of experience, held Level III/IV futsal coaching certificates, and were serving as coaches of the national first division or national teams by the time of the research. All the coaches belonging to the expert group had won at least one national or international title. The group of novices completed the Futsal Level I coach course in Portugal in the year of data collection. This study was approved by the Ethics Committee of UTAD/CIDESD (UIDB/04045/2020). The participating coaches signed the free and informed consent form, authorizing the data collection and its use for research purposes, guaranteeing their anonymity.

### Procedures

A total of 38 video clips were created to sample the attack and defensive set-piece moments of futsal. A total of 20 clips were attack set pieces (2 min of exposure): seven clips, offensive corner kicks (OfCor); seven clips, offensive sideline kicks (OfSide); six clips, offensive free kicks (OfFree) and 18 clips were defense set pieces (2 min of exposure): six clips, defensive corner kicks (DefCor); six clips, defensive sideline kicks (DefSid); and six clips, defensive free kicks (DefFree). The order of the clips for each game moment was kept constant, with the sequence of the three-game moment categories randomized across participants.

The technical footage and video clips were obtained from the Portuguese Football Federation and selected by convenience and consensus by the first and last authors (expert futsal coaches) to represent attack and defense set pieces (Roca et al., 2011). The videos were filmed from an elevated view of the field, which is a similar perspective to the videos that coaches usually use to analyze opponents. The overall videos were previously used by Portuguese Football Federation coaches for the same purpose. The participants were asked to watch the clips focusing on one of the teams, similar to how they would analyze an opponent's team. Before starting the video, they received specific instructions on whether to focus on the team's attacking or defensive play.

In this study, video clips were projected onto a screen (2.7 m × 3.6 m) using a projector (LG CineBeam LED HD 1,280 × 720) (Roca et al., 2011). The coaches were positioned two and a half meters away from the screen, allowing them to view the clips from a third-person perspective and an elevated view. When viewing the videos, all the participants used the Pupil Invisible eye-tracker system by Pupil Labs (120 Hz high-speed camera, 200 Hz eye camera), which has two built-in infrared cameras, one on each side. Infrared LEDs placed near the cameras located in the eye area were used to activate the lighting, enabling the Pupil invisible system to be used in dark environments (Tonsen et al., 2020). This setup was connected to a smartphone that recorded data relating to visual search strategies while watching the videos. The coaches were instructed to watch the video clips of the game to analyze the opponent: first by characterizing the scenes related to attack set pieces and then those related to defense set pieces. All the coaches went through a period of familiarization in which they watched all the videos one time and were given the opportunity to inquire about the data collection procedures.

### Data collection

**Figure 1:**
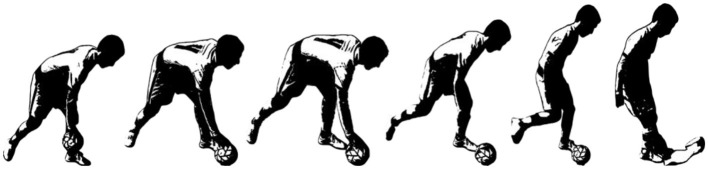
Representation of the moment the ball is placed on the ground until it leaves the performer's foot.

Data collection for set pieces in the analysis began the moment the ball was placed on the ground, and data collection was stopped the moment the ball started to move, leaving the foot of the set piece taker (Figure 1).

**Figure 2:**
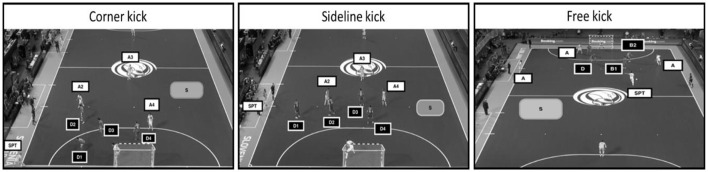
Representation of the variables included for the game moments attack and defense set pieces (free kick).

During the time in analysis, the longer fixation location, for a minimum of 100 ms of duration, and the location of the fixation were considered for analysis (Aksum et al., 2020; Casanova et al., 2022; Ballet et al., 2023). When two locations were superimposed during the fixation, both locations were registered. Thus, considering the object(s) inserted in the gaze circle, the following locations were included in the analysis (Figure 2): attacker without a ball (A), defender (D), space (S), attacker 2 (A2), attacker 3 (A3), attacker 4 (A4), defender 1 (D1), defender 2 (D2), defender 3 (D3), defender 4 (D4), set piece taker (SPT) barrier 1st line (B1) and barrier 2nd line (B2). The attackers and defenders were assigned 1–4 according to the initial distance to the ball, a strategy used by coaches to discriminate the positions of players in set pieces.

### Statistical analysis

A Shapiro–Wilk test was used to assess the data distribution. Due to the existence of a non-normal distribution of data, the comparisons between performance variables were assessed using a non-parametric test. The Mann–Whitney test was conducted to assess general differences in gaze duration for attack and defense game moments between the two groups of coaches (expert vs. novice). We also compared the gaze duration, considering the gaze location between the groups. The significance level was set at a *p*-value of ≤ 0.05. To avoid Type I errors when comparing gaze duration considering the gaze location between expert and novice coaches, multiple testing using Bonferroni correction was made, and the significance level was set at a *p*-value of ≤ 0.004. The magnitude of differences was assessed using Hedges' *g* effect sizes (≥0.2–0.5 small, ≥0.5–0.08 moderate, and ≥0.8 large effect size). The game moments (attack and defense set pieces) and the gaze location were analyzed through correspondence analysis. This is a statistical technique similar to principal component analysis, in which the common position of the categories in the distance from the center of the presentation is to be interpreted as the correlation or correspondence of the categories. This technique allows for the analysis of simple two-way tables containing some measure of correspondence between the rows and columns. The data were summarized in two-dimensional graphics, allowing the visualization of the relationships between the categorical variables game moments and gaze location. Each row and column of the contingency table represents a variable and was represented as a point in the plot. The distance between points reflects the association between variables. Points that are closer together indicate stronger associations between the correspondent variables. The correspondence analysis, through the two-dimensional graphics, provides a visual summary of the data, making it easier to understand the complex relationship between the categories of variables. Each dimension in correspondence analysis corresponds to a principal component that explains the variance obtained (i.e., inertia). Two different plots were constructed to stratify the results according to the game moments (attack and defense set pieces) and gaze location in expert and novice groups. Statistical analyses were performed using SPSS 24.0 (SPSS Inc., Chicago, IL, United States).

## Results

The analysis of the gaze duration for the attack (OfCor, OfSid, OfFree) and defense set pieces (DefCor, DefSid, DefSid) showed statistically significant differences between the groups, with moderate to large effect sizes for all the moments considered, with the exception of the defensive sideline, which revealed only a small effect. The higher values of gaze duration were observed for the group of experts than novices (Table 1). Interestingly, the group of experts revealed higher variability in gaze duration for most of the game moments than the group of novices (Table 1).

**Table 1:** Gaze duration between expert and novice coaches for each attack and defense set piece.

**Gaze duration (ms)**							
	**Experts**		**Novices**		***U***	***P***	***g***
	**Median**	**IQR**	**Median**	**IQR**			
Offensive corner kick	807.00	1,064.00	451.00	564.00	2,624.00	**0.001**	0.56
Offensive sideline kick	1,389.00	1,335.00	578.00	683.25	1,005.00	**0.001**	0.94
Offensive free kick	1,118.00	1,217.00	554.50	644.75	751.50	**0.010**	0.73
Defensive corner kick	1,034.00	800.75	463.00	573.00	818.00	**0.001**	0.74
Defensive sideline kick	1,007.00	1,377.50	664.00	836.00	775.50	**0.026**	0.41
Defensive free kick	1,380.00	1,606.75	791.00	808.50	538.50	**0.008**	0.79

IQR, interquartile range. The bold values indicate statistically significant differences.

The analysis of gaze duration (ms), considering the location, showed statistically significant differences, with moderate to large effect sizes, between groups for the variables including attacker 3 (A3), defender 3 (D3), and barrier 1st line (B1st). Only the expert coaches used the information from barrier 2nd line (B2nd line) during the time of analysis. Higher values of gaze duration, considering location, were observed for the group of experts than for novices (Table 2). As previously observed, the group of experts revealed higher variability in gaze duration considering the location than the group of novices (Table 2).

**Table 2:** Gaze duration considering the gaze location between expert and novice coaches.

**Gaze duration (ms)**							
	**Experts**		**Novices**		***U***	***P***	***g***
	**Median**	**IQR**	**Median**	**IQR**			
A	999.50	1,511.25	976.00	842.00	131.50	0.665	0.39
D	1,108.00	2,232.00	580.00	940.75	159.00	0.064	0.65
S	571.00	797.00	608.00	740.00	545.50	0.776	0.08
A2	1,104.00	1,283.00	640.00	666.50	783.00	0.011	0.57
A3	1,379.00	1,432.00	676.00	576.00	465.00	**0.001**	**0.77**
A4	1,028.00	979.00	892.00	1,924.00	180.00	0.698	0.03
D1	463.50	821.75	345.50	531.50	158.00	0.609	0.22
D2	1,063.00	1,026.00	715.00	542.25	264.50	0.008	0.61
D3	1,189.50	865.75	443.50	356.25	91.00	**0.001**	**1.31**
D4	1,079.00	1,279.00	735.00	689.50	178.00	0.136	0.49
SPT	815.00	1,097.00	308.00	317.00	138.00	0.009	1.11
B1st	1,417.50	1,279.50	369.50	414.00	27.00	**0.002**	**1.22**
B2nd	1,067.00	1,191.00	0	0	–	–	–

IQR, interquartile range. The bold values indicate statistically significant differences.

### Attack set pieces

The chi-square analysis for the attack set pieces showed a significant association between the group and gaze location (*X*^2^ = 483.04, *p* < 0.001), justifying the use of correspondence analysis. The analysis of each dimension revealed that dimension 1 explained 64.4% of the variation, and dimension 2 explained 20.6% of the variation. In total, a cumulative 84.9% of the inertia is explained by the two dimensions created. The results revealed that dimension 1 was predominantly characterized by expert offensive free kicks (45.5% of inertia) and novice offensive free kicks (30.2% of inertia), while dimension 2 was predominantly characterized by novice offensive free kicks (46.7% of inertia) and expert offensive free kicks (42.8% of inertia). Regarding the gaze location, the results revealed that dimension 1 was predominantly characterized by the attacker without the ball (A) (34.7% of inertia), the defender (D) (21.2% of inertia), the barrier 1st line (B1st) (13.4 of inertia), attacker 2 (A2) (8% of inertia), and attacker 3 (A3) (7% of inertia). Dimension 2 was predominantly characterized by the attacker without ball (A) (31.1% of inertia), defender (D) (28.7% of inertia), space (S) (13.8% of inertia), and barrier 1st line (B1st) (11% of inertia).

**Figure 3:**
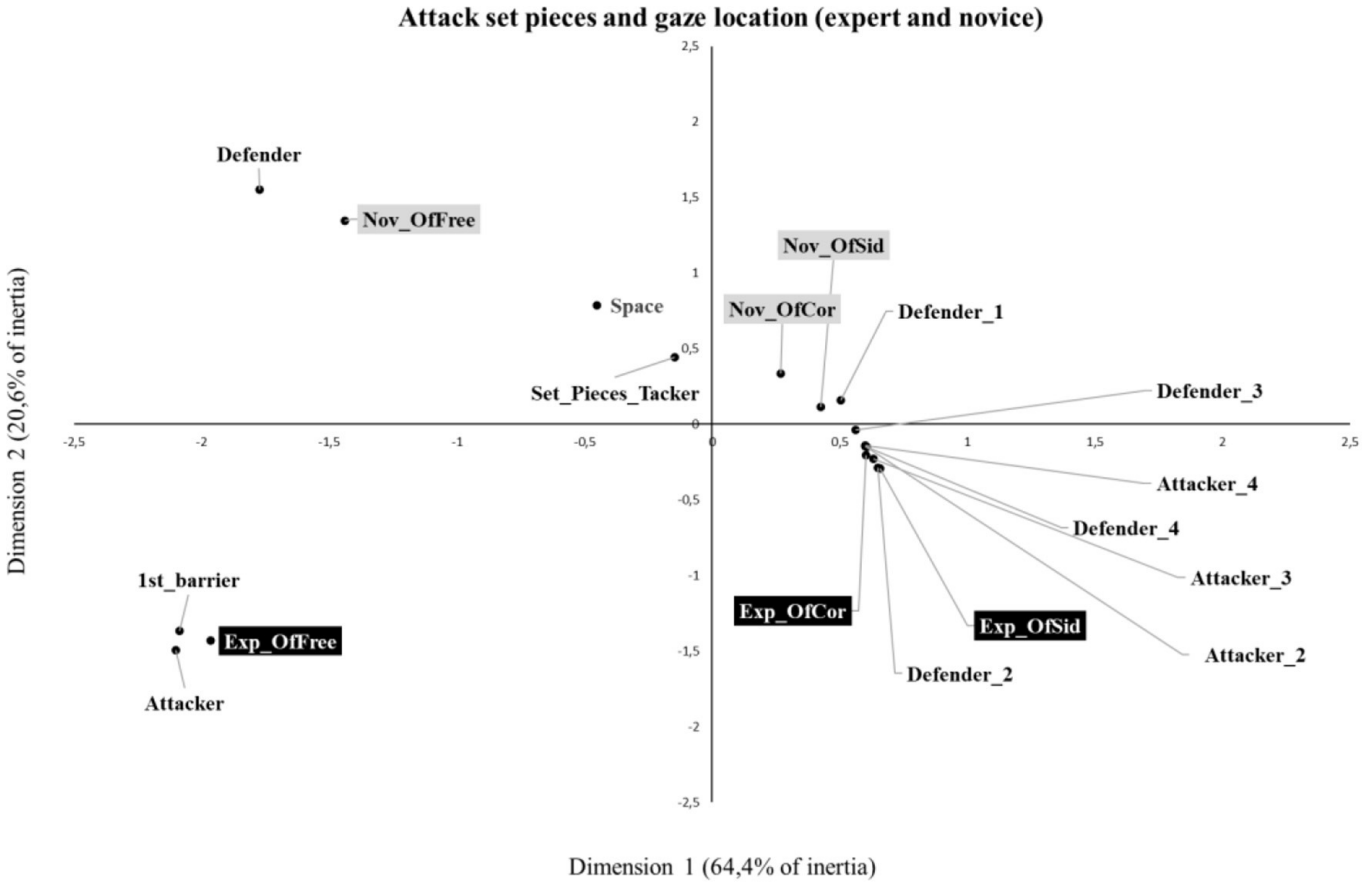
Biplot from the correspondence analysis of game moments (attack set pieces) and gaze location.

By examining the biplots of attacking set pieces (Figure 3), the expert and novice coaches were always represented in different quadrants for the same set piece moment, revealing a distinct relationship with gaze location variables, particularly explained by variations in dimension 2. While the analysis of offensive free kicks by experts (Exp-OfFree) is represented in the left lower panel, the analysis of offensive free kicks by novices (Nov–OfFree) is represented in the left upper panel. Accordingly, different associations were observed between gaze location variables. The offensive free kicks of experts (Exp-OfFree) revealed some correspondence with the gaze locations attacker without a ball (A) and barrier 1st line (B1), while the offensive free kicks of novices (Nov–OfFree) revealed some correspondence with the gaze locations defender (D), space (S), and set piece taker (SPT).

The analysis of the offensive corner and sideline of experts (Exp-OfCor and Exp-OfSid) is represented in the lower right panel, while the offensive corner and sideline of novices (Nov-OfCor and Nov-OfSid) are represented in the upper right corner. However, the distance between them was small, not allowing them to clearly distinguish the preferential information that is used by each one.

### Defense set pieces

The chi-square analysis for the defense set pieces showed a significant association between the group and gaze location (*X*^2^ = 457,21, *p* < 0.001), justifying the use of correspondence analysis. The analysis of each dimension revealed that dimension 1 explained 61.2% of the variation, and dimension 2 explained 19.8% of the variation. In total, a cumulative 81.1% of the inertia is explained by the two dimensions created. The results revealed that dimension 1 was predominantly characterized by expert defense free kicks (55.7% of inertia) and novice defense free kicks (17.6% of inertia), while dimension 2 was characterized by novice defense free kicks (66.2% of inertia) and expert defense free kicks (25.4% of inertia). Regarding the gaze location, the results revealed that dimension 1 was predominantly characterized by an attacker without ball (A) (26.9% of inertia), defender (D) (21.9% of inertia), and barrier 1st line (21.3% of inertia). Dimension 2 was predominantly characterized by an attacker without a ball (A) (37.8% of inertia), a defender (27.7% of inertia), and a set-piece taker (SPT) (22.8% of inertia).

**Figure 4:**
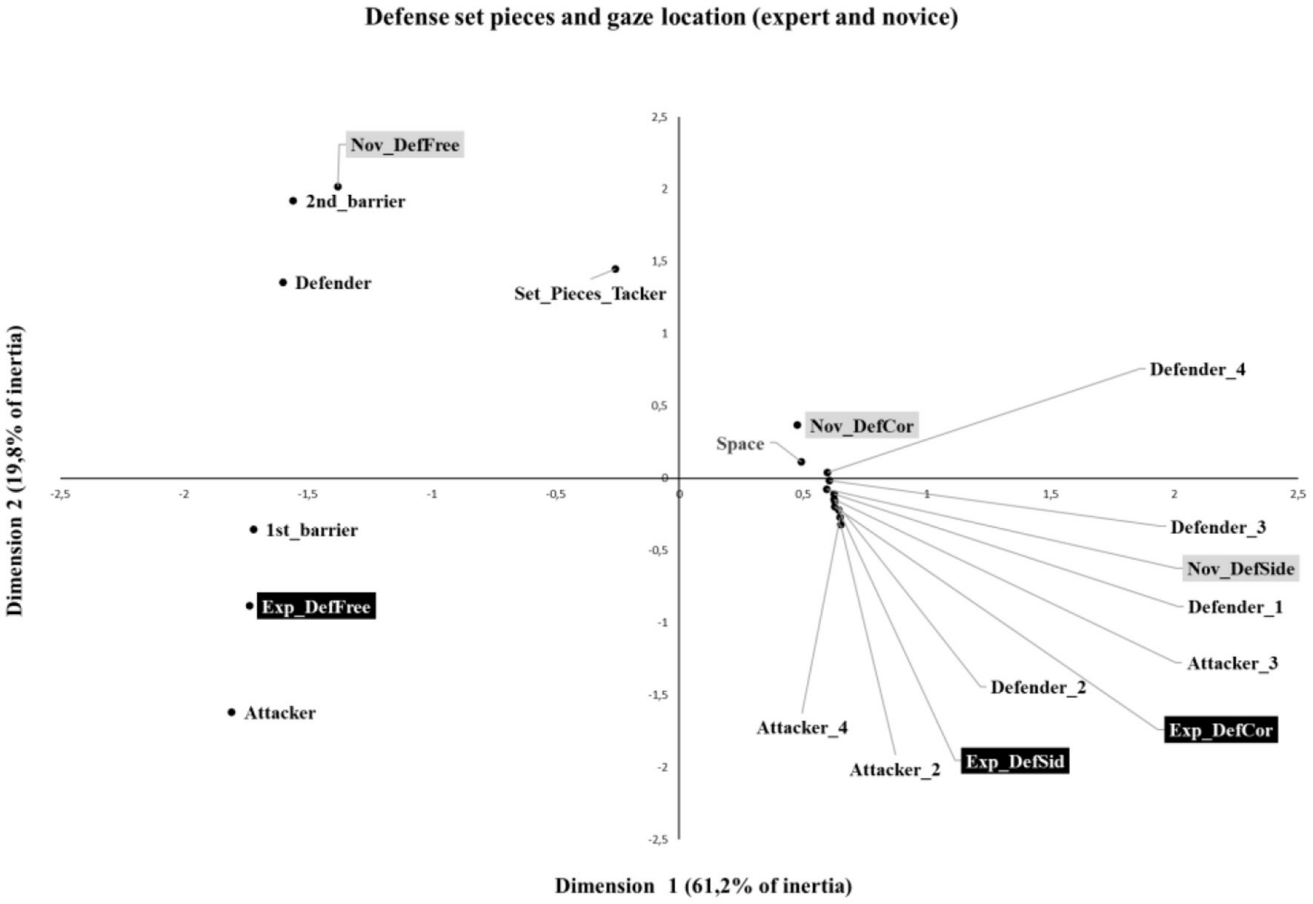
Bi-plot from the correspondence analysis of game moments (defense set pieces) and gaze location.

By examining the biplots of defensive set pieces (Figure 4), the expert and novice coaches were always positioned in different quadrants for the same set piece moment, revealing a distinct relationship with gaze location variables, particularly explained by variations in dimension 2. The analysis of defensive free kicks of experts (Exp-DefFree) is represented in the lower left panel, while novices (Nov–DefFree) appear in the upper left panel. Accordingly, different associations were observed with gaze location variables. The defensive free kicks by experts (Exp-DefFree) revealed some correspondence with the gaze locations attacker without a ball (A) and barrier 1st line (B1), while the defensive free kicks by novices (Nov–DefFree) revealed some correspondence with gaze locations defenders (D), barrier 2nd line (B2), space (S), and set piece takers (SPTs).

The analysis of the defensive corner and sideline of experts (Exp-DefCor and Exp-DefSid) is displayed in the lower right panel, while the defensive corner and sideline of novices (Nov-DefCor and Nov-OfSid) are shown in the upper right corner. However, the distance between them was small, hindering the clear differentiation of the preferential information that is used by each group.

## Discussion

This study aimed to describe the fixation location and the time of the longer fixation of expert and novice futsal coaches before the ball was in play in futsal set pieces. The results partially confirm the hypothesis that expert coaches present longer fixations at different locations than novice coaches. The gaze duration presented significant differences between expert and novice coaches for all the set pieces in the analysis. Expert coaches tend to fix more time on the gaze in attacker 3 (A3), defender 3 (D3), barrier 1st line (B1st), and barrier 2nd line than novices. Moreover, variations in the gaze location between expert and novice coaches were only clear for attack and defense free kicks. The correspondence between attack/defense corner and sideline kicks did not show variations in the correspondence according to the level of expertise.

The current results reinforced the idea that the visual information that sustains understanding of each game moment is specific and needs to be considered in the coaches' development process (Rodrigues et al., 2024). Particularly, in the analysis of free kicks, novice coaches could be aware of the information that they pick up before the ball is in play to anticipate the players' possibilities for action (Araújo et al., 2009). This result highlights the information used to support their decision and contributes to further understanding of the key information that should be considered for each game moment (Mann et al., 2007).

In the context of this study, the set pieces analysis was considered to understand how coaches explore visual information before the ball is in play, i.e., during the time the ball is placed on the ground until it leaves the set piece taker's (SPT) foot in set pieces moments. Therefore, expert coaches revealed longer fixations, often occurring at the end of the set piece. The longer the observation of specific information by the expert coaches in comparison with the novice, even when the ball is stopped, could mean the great ability of coaches to be attuned to the key information of the context (Withagen et al., 2012). In other words, speculatively, the higher values of gaze duration verified by the expert coaches could be the result of a clear knowledge of the spatial-temporal relationships that promote offensive or defensive advantage in game moments of set pieces (Araújo et al., 2009). The observed higher variability in gaze duration values for expert coaches could result from the individual strategies of exploration of opportunities for action according to their own knowledge and game model.

Before the ball is in play, expert coaches tend to fixate more time on attacker 3 (A3), defender 3 (D3), barrier 1st line (B1st), and barrier 2nd line (B2nd) when compared to novices. In set pieces, particularly in the corner and sideline kicks, the attacker and defender 3 are usually the players that directly or indirectly create space to open passing and shooting lines for themselves or other players. Thus, understanding their angular relationship and the space between attacker and defender 3 in relation to the goal is fundamental before the ball is in play to understand the spaces created for shooting (Corrêa et al., 2014; Vilar et al., 2014). In free kicks, expert coaches tend to fixate more time on the barrier 1st line (B1st) and barrier 2nd line (B2nd) compared to novices. This information could be used to understand the number of players in the barrier and their relative position on the field in relation to the ball and goal, and it is crucial in understanding the numerical balance between attackers and defenders in relation to the goal. This understanding is fundamental for prospectively assessing players' possible passing and shooting lines in the field and anticipating possible actions, which is characteristic of expert coaches (Savelsbergh et al., 2002; Mann et al., 2007).

In the analysis of attack and defense set pieces, correspondence analysis shows different visual search strategies and, consequently, gaze locations for each game moment. The variance of dimensions 1 and 2 (i.e., inertia) was almost explained by expert and novice free kicks for both attack and defense, revealing major differences between expert and novice coaches. In contrast, the variance explained by the corner and sideline kicks was too small. This means that there is some correspondence between the gaze locations of expert and novice coaches on corner and sideline kicks, while free kicks present a variation in correspondence between the groups and gaze locations.

The analysis of offensive and defensive free kicks revealed that expert coaches tend to preferentially focus on the informational variables of the barrier's 1st line and attackers without the ball. In contrast, novice coaches, during offensive plays, focus on the variables including defender, space, and set piece taker, while in defensive play, their attention is drawn to the barrier 2nd, defender, space, and set piece taker. In line with previous research, experts appear to be more selective in the informational variables considered to understand the possibilities for action during free kicks compared to novices (Mann et al., 2007). These results suggest that novice coaches may face greater challenges in identifying relevant cues necessary to understand the game's potential actions effectively (Abernethy and Russell, 1987; Abernethy, 1990; Mann et al., 2007; Mitchell et al., 2020).

Regarding offensive and defensive corners and sidelines, novice coaches revealed a close correspondence with the informational variable defender 1. Expert coaches have a close correspondence with the informational variables including attacker 2, attacker 3, attacker 4 and defender 2, defender 3, and defender 4. It is not possible to assume that there is a clear distinction between their visual strategies. Due to the small space in which such moments occur, further research is required to understand whether the lack of differences was related to similarities in the visual search of expert and novice coaches or to methodological issues regarding the identification of the gaze location in the video. Besides the differences in visual strategies between expert and novice coaches identified in this study, questions such as “Are there other relevant sources of information that coaches are looking for?” arise. These questions cannot be answered due to the technological limitations of the device used in this research, but there is a need in the future to capture the real intentions of the expert coaches that guide their gaze, visual search strategies, and their fine perception (Wood et al., 2023). Further research using qualitative methods could be particularly relevant to understanding the intentions and knowledge underpinning expert coaches' behaviors. Furthermore, some research studies need to be conducted in coach education courses to understand the effects of using video analysis as a learning strategy to guide novice coaches' visual search strategies. Despite the results obtained, we acknowledge that the participation of the reduced number of coaches is a limitation of this study. Moreover, for comparison purposes, data collection was carried out using videos and not in the futsal game setting. Further research is required to understand the major differences between coaches' visual search behaviors in analyzing opponent teams during the competition or in the video.

## Conclusion

Based on the hypotheses, we can conclude that experts present longer fixations at different locations than novice coaches, particularly for free kicks. To the best of our knowledge, this was the first study to attempt to investigate the visual search strategies of coaches in set pieces, particularly before the ball is in play. These results can improve the understanding of the critical information that may underlie attack and defense set pieces in Futsal. In free kicks, coaches should be particularly concerned about the relationship between attacker and defender 3 and the barrier 1st and 2nd line positions. In corner and sideline kicks, coaches should be particularly aware of the relationship between attackers' and defenders' positions. This information could be used by novice coaches to develop visual search strategies for set pieces before the ball is in play, improving their capacity for anticipation and decision-making during the game.

## Data availability statement

The raw data supporting the conclusions of this article will be made available by the authors, without undue reservation.

## Ethics statement

The studies involving humans were approved by Ethics Committee of UTAD/CIDESD. The studies were conducted in accordance with the local legislation and institutional requirements. The participants provided their written informed consent to participate in this study.

## Author contributions

MR: Conceptualization, Formal analysis, Investigation, Methodology, Software, Writing – original draft, Writing – review & editing. NL: Conceptualization, Investigation, Methodology, Supervision, Validation, Writing – original draft, Writing – review & editing. JR: Formal analysis, Investigation, Methodology, Writing – review & editing. JS: Conceptualization, Validation, Writing – review & editing. DA: Conceptualization, Validation, Writing – review & editing. BT: Conceptualization, Formal analysis, Investigation, Methodology, Resources, Supervision, Visualization, Writing – original draft, Writing – review & editing.
